# Modulation of Hippocampal Sharp-Wave Ripples by Behavioral States and Body Movements in Head-Fixed Rodents

**DOI:** 10.1523/ENEURO.0012-25.2025

**Published:** 2025-07-23

**Authors:** Alain Rios, Minori Usui, Yoshikazu Isomura

**Affiliations:** Department of Physiology and Cell Biology, Graduate School of Medical and Dental Sciences, Institute of Science Tokyo, Tokyo 113-8519, Japan

## Abstract

Hippocampal sharp-wave ripples (SWRs) are critical events implicated in memory consolidation, planning, and the reactivation of recent experiences. Under freely moving conditions, a well-established dichotomy exists: hippocampal networks predominantly generate theta oscillations during periods of reward pursuit (preparatory behaviors) and exhibit pronounced SWR activity once the reward is achieved (consummatory behaviors). Here, it was examined how SWRs are modulated by reward delivery and small movements in head-fixed rats. Contrary to the canonical view established in freely moving settings, the results revealed that the dominant and more enduring effect was a sustained suppression of SWR activity immediately following water delivery. Moreover, even minor, localized movements (such as whisking or body adjustments) decreased SWR occurrence, demonstrating that hippocampal ripple generation is highly sensitive to motor engagement, irrespective of reward timing. Such movement-induced suppression of ripples persisted during both sleep-like states and quiet wakefulness, suggesting that while large-scale brain states modulate the overall likelihood of SWR generation, local motor-related influences exert a state-independent inhibitory effect on hippocampal ripples. These results show that SWR modulation by behavioral states and body movements is more context dependent than previously appreciated.

## Significance Statement

Small body movements robustly suppress hippocampal sharp-wave ripples across all behavioral states in head-fixed rodents. This reveals a highly context-dependent modulation of SWRs, redefining our understanding of hippocampal ripple dynamics during rest and water consumption.

## Introduction

The hippocampus is integral to learning and memory, functioning within a complex network of brain regions including the prefrontal cortex, medial entorhinal cortex, and frontal cortex (Harvey et al., 2023). Among the various electrophysiological phenomena observed in the hippocampus, sharp-wave ripples (SWRs) are prominent. These high-frequency, high-amplitude oscillations occur primarily in the CA3 and CA1 regions and are associated with the reactivation of previously active neural ensembles. This reactivation is thought to contribute to memory consolidation, decision-making, planning, and creative thinking (Ji and Wilson, 2007; Buzsáki, 2015). Moreover, disruptions in SWR activity have been implicated in a range of neurological and psychiatric disorders, including epilepsy, schizophrenia, and Alzheimer's disease (Suh et al., 2013; Zhen et al., 2021).

Extensive research has demonstrated that SWRs occur most frequently during states of immobility, sleep, and consummatory behaviors (Singer and Frank, 2009; Buzsáki, 2015). Consummatory behaviors fulfill basic physiological needs such as eating, drinking, resting, and defecating (Ball and Balthazart, 2008). Historically, SWRs were thought to primarily facilitate learning and memory consolidation once these needs had been met, supporting the retrospective processing of experiences after immediate drives were satisfied.

However, recent investigations have revealed a more nuanced relationship between SWRs and consummatory behaviors. For instance, recent reports demonstrated a decrease in SWR activity during the intake of water rewards (Klee et al., 2021; Sakairi et al., 2025), suggesting that these dynamics may be closely modulated by the animal's current behavioral state. A critical distinction in these studies is whether the animals were head-fixed or allowed to move freely, indicating that SWR patterns can be differentially regulated based on experimental context and the state of motion. These findings underscore the importance of behavioral and environmental factors in shaping hippocampal dynamics.

To further examine how SWRs relate to distinct behavioral states, experiments were conducted in head-fixed rats, with simultaneous tracking of their movements and recording of hippocampal activity. First, the SWR frequency surrounding reward delivery events (randomly provided water) was evaluated, and subsequently, the relationship between changes in the rats’ activity levels and SWR occurrence was assessed throughout the entire session. Through this approach, a more comprehensive understanding of the interplay among SWRs, behavioral states, reward processing, and body movements is provided, offering new insights into how hippocampal neural activity aligns with different behavioral states.

## Materials and Methods

### Animals and surgery

All experiments were approved by the Animal Care and Use Committee of Institute of Science Tokyo (A2023-116) and were performed in accordance with the Fundamental Guidelines for Proper Conduct of Animal Experiment and Related Activities in Academic Research Institutions (MEXT, Japan) and the Guidelines for Animal Experimentation in Neuroscience (Japan Neuroscience Society). All surgical procedures were performed under appropriate isoflurane anesthesia, and all efforts were made to minimize suffering (Nonomura et al., 2018; Rios et al., 2023).

Four wild-type Long–Evans rats (8–10 weeks old; 210 ± 35 g; male, 2; female, 2) were kept in their home cages under an inverted light schedule (lights off at 9:00 A.M.; lights on at 9:00 P.M.). The rats were briefly handled by the experimenter (10 min, twice) in advance. For head-plate (CFR-2, Narishige) implantation, the animals were anesthetized with isoflurane (4.5% for induction and 2.0–2.5% for maintenance, Pfizer) using an inhalation anesthesia apparatus (Univentor 400 anesthesia unit, Univentor) and were placed on a stereotaxic frame (SR-10R-HT, Narishige). Lidocaine (AstraZeneca) was administered around the surgical incisions. Reference and ground electrodes (Teflon-coated silver wires, A-M Systems; 125 μm diameter) were implanted above the cerebellum. During anesthesia, body temperature was maintained at 37°C using an animal warmer (BWT-100, Bio Research Center). Analgesics and antibiotics were applied postoperatively, as required (meloxicam, 1 mg/kg, s.c., Boehringer Ingelheim; gentamicin ointment, 0.1%, MSD). A second surgery was performed for later electrophysiological recordings. We made two tiny holes (1.0–1.5 mm in diameter) in the skull and dura mater above the dorsal hippocampi bilaterally (3.8 mm posterior and ±2.0 mm lateral from the bregma). The skull surface and holes were immediately covered with the antibiotic ointment and silicon sealant until the recording experiments.

After full recovery from surgery (3–7 d later), the rats had brief acclimation sessions to the head-fixed apparatus. Specifically, each rat was placed in the recording box and head-fixed for 10 min on two different occasions, mimicking the conditions of the actual recording. This step ensured that the animals gradually adapted to head fixation, minimizing potential stress. The rats had *ad libitum* access to water during weekends, and during the rest of the week, they obtained water only during the recording session. Additionally, agar was given to the rats in their home cage to maintain them at >85% of original body weight (Isomura et al., 2009; Kimura et al., 2012; Soma et al., 2017).

### Electrophysiological recording

Extracellular multichannel recordings of local field potentials (LFPs) and single-neuron spike activity were obtained from the hippocampal CA1 area. In two rats, we simultaneously recorded from the parietal cortex located above the CA1 recording sites (∼1.2 mm below the cortical surface). Two 32-channel silicon probes (ISO-3x-tet-lin with seven tetrode-like arrangements and four vertically aligned channels on three shanks; NeuroNexus Technologies) were inserted vertically into the left CA1 or the parietal cortex, supported by a 2% agarose-HGT (Nacalai Tesque) gel layer on the brain surface.

Recording depth was determined to detect ripple oscillations within the stratum pyramidale (s.p.) of CA1 (Isomura et al., 2006), typically at the second tetrode from the probe tip. Thus, the upper tetrodes were located in the stratum oriens (s.o.) and the lower (tip) tetrodes in the stratum radiatum (s.r.). After the silicon probe was lowered to the appropriate depth, it was left in place for 1 h before initiating the data collection. During this period, the rat remained head-fixed under the same conditions used for recording, allowing additional habituation and minimizing any acute stress response due to handling or probe insertion. In the final recording session, probes were coated with the fluorescent dye DiI (DiIC18(3), PromoKine) to visualize probe tracks post hoc.

Neuronal activity was amplified (32-channel main amplifier: FA-32, Multi Channel Systems; final gain: 2000; bandpass filter: 0.5 Hz–10 kHz) through a 32-channel head-stage preamplifier (MPA 32I, Multi Channel Systems; gain: 10). Signals were digitized at 20 kHz using a 32-channel hard-disk recorder (LX-120, TEAC).

### Histology

Following the final recording sessions, rats were deeply anesthetized with urethane (2–3 g/kg, i.p.) and perfused transcardially with cold saline followed by 4% paraformaldehyde in 0.1 M phosphate buffer (PB). The brain was then postfixed and sectioned coronally at 50 µm using a vibratome (VT1000S, Leica). Fluorescent DiI-labeled probe tracks and hippocampal CA1 lamination were examined under a fluorescence microscope (BX51N, Olympus).

### SWR, sleep spindles, and LFP delta power analysis

SWR detection followed standard threshold criteria (Csicsvari et al., 2000; Isomura et al., 2006; O'Neill et al., 2006). Briefly, multichannel signals were downsampled to 1 kHz and bandpass filtered in the ripple range (150–250 Hz). The signal envelope was obtained using the Hilbert transform, and SWR events were defined as epochs exceeding 4 standard deviations from the mean (we excluded such events exceeding 9 standard deviations, considering artifacts) for at least 50 ms. The amplitude and peak time of each SWR were defined as the peak amplitude of the enveloped signal in the ripple-band LFP. When SWRs overlapped across multiple channels, the event with the largest peak amplitude was selected for analysis. Artifacts and noise were identified and removed by comparing signals from channels with lower ripple counts and by visual inspection independent of behavior-related information.

For cortical delta power analysis, the LFP signal was bandpass filtered at 0.5–4 Hz and the envelope was estimated via the Hilbert transform. Periods of delta-on activity were defined as those exceeding 4 standard deviations for at least 200 ms (Norman et al., 2021).

Sleep spindles were detected by applying an 8–16 Hz bandpass filter to raw LFP signal. The envelope of the signal was extracted by computing the Hilbert transform of the bandpass-filtered signal. A time window was classified as a spindle if the upper envelope exceeded a threshold of 2.5 SD above the mean once and the lower envelope crossed a threshold of 1.5 SD below the mean for >300 ms (Wang et al., 2024).

### Video recording and analysis

Three cameras (Basler ace PoE camera acA1440-73 B/W, Edmund) were positioned at front, diagonal, and overhead angles to record the animals’ movements. The initial frame rate (50 frames/s) was frame blended and interpolated to 30 frames/s for analysis using FFmpeg software (FFmpeg team). Movement detection was based on a pixel-wise absolute difference (PWAD) calculation (Soma et al., 2019):

$$\mathrm{PWA}D_{i}=\mathrm{Fram}e_{i}(x,y)-\mathrm{Fram}e_{i-1}(x,y),$$

where Frame_i_ represents the *i*th video frame. First, PWAD averages of all frames were obtained for the entire session. Next, regions showing higher levels of movement were identified visually as regions of interest (ROIs), including the orofacial region (tongue, jaw, whiskers, nose), forelimbs, body, and tail. The PWAD values within each ROI were then averaged to generate a continuous pixel-wise difference average (PWDA) per ROI. Movement events were defined as periods where the PWDA exceeded 2.5 standard deviations above the baseline (Musall et al., 2019).

For every pair of ROIs the Pearson’s correlation coefficient (*R*) was calculated across the full session. A symmetric distance matrix,

$$D_{\mathrm{ij}}=1-R_{\mathrm{ij}},$$

was constructed and submitted to agglomerative hierarchical clustering with average-linkage criterion (SciPy cluster.hierarchy.linkage, method = “average”). The resulting dendrogram was cut at the cophenetic distance that maximized the inconsistency coefficient gap (Fig. 4*K*). Because licking events were temporally sparse and mechanically distinct, tongue movements that coincided with water-port contact were tagged separately as Licking.

### Spike analysis

Offline spike sorting was performed to isolate single units from each tetrode. Spike candidates were initially detected and clustered using EToS (Takekawa et al., 2010, 2012). Manual refinement of these clusters was performed using Klusters and NeuroScope (Hazan et al., 2006), ensuring that single-neuron clusters had a clear refractory period (>2 ms) in autocorrelograms and no refractory period contamination in cross-correlograms with other clusters. Single-neuron clusters were included if they contained at least 250 spikes in total (Kawabata et al., 2020; Mitani et al., 2022).

### Experimental design and statistical analyses

Electrophysiological CA1 data were obtained from 66 recording sessions across four rats. In a subset of 12 sessions from two rats, we additionally recorded the activity from deep layers of the parietal cortex to evaluate LFP delta power. Each session lasted ∼90 min, during which water (for the sake of simplicity we will call it reward) was delivered through a spout in front of the animal's mouth at a random time among a uniform distribution from 140 to 220 s, with no external cues. Each delivery consisted of three 5 µl drops administered by a micropump. SWR activity was aligned to both water delivery events and ROI-defined movement events to construct perievent time histograms (PETHs).

SWR frequency was normalized (*z*-score) on a session-by-session basis using a baseline window (−5 to −2.5 s relative to the event). Two test windows were compared against this baseline: 0 to +0.5 s and 0 to +2.5 s relative to water delivery or movement events. Comparisons were conducted on a trial-by-trial basis within and across sessions using Wilcoxon signed-rank test. Correlations between cortical LFP delta power and CA1 SWR activity were assessed with Pearson's correlation, and significance was evaluated using a *t* test on the correlation coefficients.

To examine the contribution of cortical delta power and ROI-specific movements to hippocampal ripple probability, we implemented an L1-regularized logistic regression analysis using binarized time series from individual recording sessions. SWR occurrence was first binned into 100 ms intervals across the full session duration, with binary indicators (1 = SWR detected, 0 = no SWR) assigned per bin. Two additional binary regressors were computed for each bin: a Delta regressor marked whether a delta event occurred in the preceding 2 s window, and a Movement regressor indicated the presence of ROI-specific movement within the preceding 1 s. An interaction term (Delta × Movement) was included to assess potential synergistic or antagonistic effects. This balanced dataset was used to fit a logistic regression model predicting ripple presence (dependent variable) from the three predictors (Delta, Movement, Delta × Movement), with a constant intercept term. L1 regularization (lasso penalty) was applied to encourage sparse solutions and reduce overfitting (*α* = 0.1). Regression coefficients for each predictor were extracted and interpreted across sessions to quantify the direction and consistency of ripple modulation by delta oscillations and ROI-specific movements.

To account for repeated measurements and avoid pseudoreplication, we quantified the movement-related change in ripple incidence for every recording subsession as follows: Δ = SWR_Test_ − SWR_Baseline_. The analysis set comprised 66 subsessions obtained from four rats (median, 16.5 sessions; range, 10–21). A linear mixed-effects model, Δ_ij_ = *β*_0_ + *u*_0j_ + *ε*_ij_, was then fitted with a fixed intercept *β*_0_ (population mean Δ) and a random intercept *u*_0j_ capturing between-rat variability [*u*_0j_ ∼ *N* (0, *σ*^2^_rat_)]. Models were estimated via restricted maximum likelihood. Significance of *β*_0_ was evaluated with a two-tailed Wald *t* test (*α* = 0.05); a significantly negative *β*_0_ indicates robust suppression of SWR incidence during movement after accounting for inter-animal variability (Pinheiro and Bates, 2000; Lazic, 2010).

All data are expressed as mean ± SD, and *n* indicates the sample size. Line plots and shaded areas represent mean ± SEM. All statistical analyses were performed with MATLAB's Statistics and Machine Learning Toolbox (The MathWorks) and Python (NumPy 1.26.4, pandas 2.2.2, SciPy 1.12.0, statsmodels 0.15.0). Differences were considered significant at *p* < 0.05. Blinding and randomization were not performed.

## Results

### Behavioral states and movement quantification during head fixation

**Figure 1.**
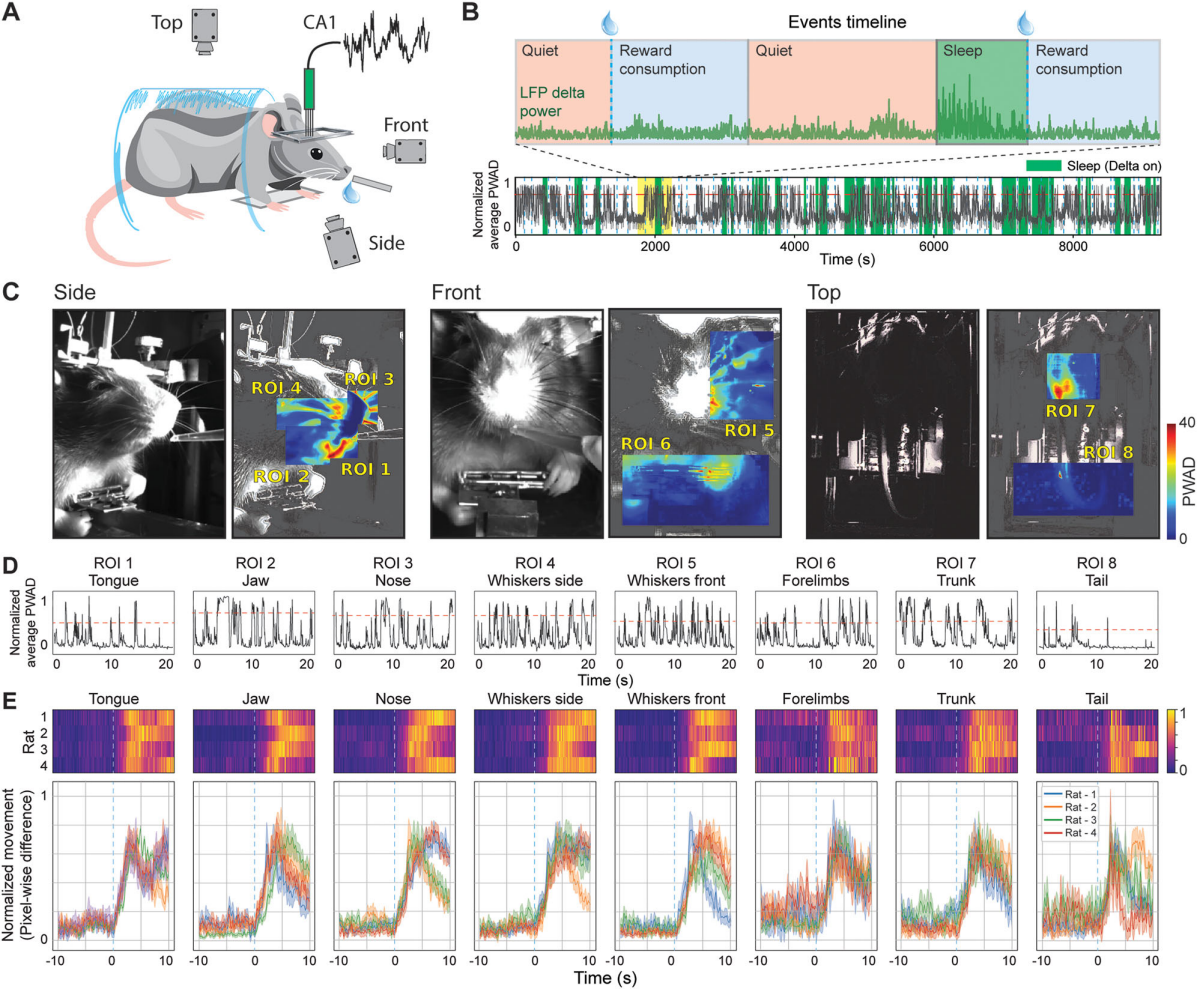
Experimental setup, behavioral-state classification, and movement quantification. ***A***, Schematic illustration of the head-fixed experimental configuration. A rat is shown with a recording electrode targeting the hippocampal CA1 region, while three cameras (side, front, top) simultaneously capture the animal's behavior. Water rewards are delivered via a spout positioned near the rat's mouth. ***B***, Representative timeline of a recording session showing the full duration with periods of quiet, reward consumption, and putative sleep (green shading, “Delta on”) identified by cortical LFP delta power (green trace). The blue drops above the timeline mark random water-reward deliveries. The black trace illustrates a representative movement signal from one region of interest (ROI 4: whiskers side), with the red dotted line indicating the threshold used for movement-event detection. The yellow segment highlights the magnified window shown above. ***C***, Example frames from each camera angle (side, front, top) used to define movement ROIs. Left panels show raw video images; right panels display heat maps of pixel-wise differences highlighting areas of increased movement. Labeled ROIs are as follows: ROI 1, tongue; ROI 2, jaw; ROI 3, nose; ROI 4, whiskers (side); ROI 5, whiskers (front); ROI 6, forelimbs; ROI 7, trunk; and ROI 8, tail. ***D***, Representative traces of pixel-wise difference amplitude (PWDA) for each ROI, illustrating how thresholding (red dashed line) is applied to detect movement events. ***E***, Top, Color maps of normalized movement event frequency aligned to reward delivery (time zero) for each ROI, with each horizontal row representing data from a single rat. Bottom, Average perievent time histograms (PETHs) for each ROI, depicting the normalized movement frequency across rats. Shaded areas indicate SEM, and vertical dashed lines mark the time of reward delivery. Please see Extended Data Figure 1-1 for additional data.

We recorded hippocampal CA1 LFPs together with high-resolution video from three camera angles in head-fixed rats to assess behavioral states and small movements during reward delivery (Fig. 1*A*, Movie 1). The placement of the recording electrode and the camera orientations allowed precise tracking of orofacial and body movements under near-immobile conditions, ensuring that detected movements were not confounded by locomotion.

10.1523/ENEURO.0012-25.2025.f1-1 Figure 1-1 Within- and across-session progression of movement events. (A) Representative data from three rats illustrating the time course of movement events for each region of interest (ROI) within a single session. Each panel shows the event count over time for one ROI, allowing visualization of how movement frequency changes throughout the session. (B) Example progression of average movement event frequency (events/s) across multiple recording sessions for a single rat. Each trace corresponds to one ROI, revealing the relative stability or variability of movement patterns as the sessions progress. Download Figure 1-1, TIF file.

**Movie 1.** Quiet resting. Twenty-four-second recording centered on a single water-reward delivery (time 0 s). The top left composite displays simultaneous side-, front-, and top-camera views of the head-fixed rat. The bottom left panel shows the hippocampal LFP filtered at 150–250 Hz in real time. Right panels plot *z*-scored movement-amplitude traces for the tongue, jaw, nose, whiskers front, and forelimbs ROIs. [View online]

Behavioral epochs were defined based on both experimental events and physiological signals. Specifically, water-reward delivery events were interleaved with intervals of relative immobility, and fluctuations in cortical LFP delta power were used to classify distinct arousal states (Fig. 1*B*). Epochs characterized by high delta power were regarded as putative sleep, whereas periods of low delta power corresponded to quiet wakefulness and active reward consumption (Fig. 4). Although our analysis is not based on discrete trials, we quantified the continuous transitions between these epochs across 66 sessions from four rats.

To assess the animals’ movements in detail, pixel-wise absolute difference (PWAD) analyses was performed on the video frames (Fig. 1*C*; see Materials and Methods). ROIs were defined for various body parts, including the tongue, jaw, nose, whiskers, forelimbs, trunk, and tail, capturing reward-locked changes in movement (Fig. 1*D*). Across all three camera views, these analyses revealed pronounced orofacial and forelimb movements tightly coupled to water-reward delivery, as well as smaller adjustments in body posture and whisking behavior during quiet periods or transitions into rest or sleep states.

Quantitative analysis (Fig. 1*E*) confirmed that the frequency of orofacial movements (e.g., tongue, jaw, and nose) increased sharply around reward events (tongue, 9.15 ± 1.23 Hz; jaw, 8.45 ± 1.79 Hz; nose, 1.47 ± 0.97 Hz; whiskers side, 1.45 ± 0.82 Hz; whiskers lat, 1.35 ± 0.80 Hz), reflecting the animals’ anticipatory and consummatory responses. In contrast, movements of other body parts (trunk, 0.83 ± 0.54 Hz; tail, 0.71 ± 0.55 Hz) exhibited distinct temporal profiles, often peaking at different times or showing more gradual changes over the trial epoch. Movements remain constant along individual and across sessions (Extended Data Fig. 1-1). Together, the consistency of movements across animals around reward-related events and ROI-based analyses provides a detailed behavioral framework against which we will interpret the temporal modulation of SWR activity in subsequent sections.

### Modulation of SWR activity during consummatory behavior

**Figure 2.**
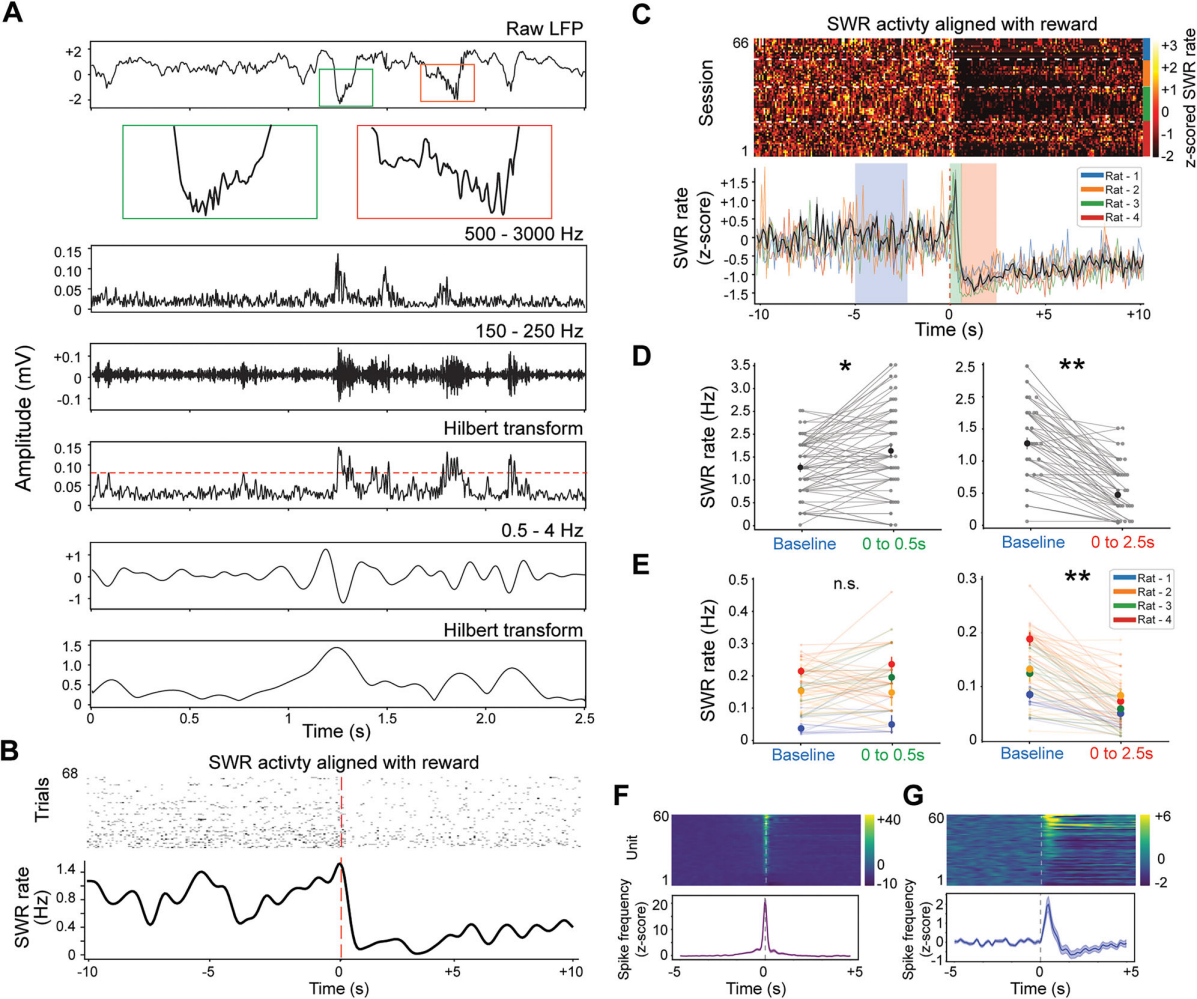
Modulation of hippocampal SWR activity and CA1 spiking by reward events. ***A***, Representative example of SWR and delta-related epoch detection. Top, Raw CA1 LFP trace with two magnified segments (green and red boxes) highlighting transient high-frequency ripple oscillations characteristic of SWRs. Middle, Bandpass-filtered signals (500–3,000 and 150–250 Hz) and their Hilbert transform envelopes. The red dashed line indicates the SWR detection threshold (4 standard deviations). Bottom, Representative cortical 0.5–4 Hz trace and its Hilbert transform envelope used to identify delta-on and delta-off periods. ***B***, Single-session example showing SWR occurrences aligned to reward delivery. Top, Raster plot of SWR events from multiple trials. Bottom, PETH of SWR frequency, illustrating a phasic decrease in SWR activity immediately following the reward (dashed vertical line). ***C***, Population-level SWR activity aligned to reward delivery. Top, Heat map of *z*-scored SWR frequency, with each row representing one session. The white dotted lines divide sessions by rat, indicated by the color bar on the right. Bottom, Mean population PETH (gray trace) with shaded SEM, overlaid with individual rat PETHs in color. Blue shading represents baseline, and the green and red shaded regions highlight short-latency (0–0.5 s) and extended (0–2.5 s) postreward windows, respectively. ***D***, Representative session showing trial-by-trial changes in SWR rate from baseline to the short-latency (left) and extended (right) postreward periods. Dots represent individual trials; colored dots indicate the mean. Asterisks denote significant differences (*p < 0.05; **p < 0.01; Wilcoxon signed-rank test). ***E***, Population-level comparisons of SWR frequency changes in short-latency (left) and extended (right) postreward intervals relative to baseline. Each rat is shown in a different color (**p < 0.01; Wilcoxon signed-rank test). ***F***, Cross-correlograms of single-unit firing relative to SWR maxima, showing all recorded neurons (top) and the average (bottom). ***G***, Population spike firing rates of CA1 neurons aligned to reward delivery (time zero). The top panel shows the color-coded spike frequency across units, while the bottom panel displays the average firing rate change. Vertical dashed lines denote the time of reward delivery.

Next, it was examined how hippocampal SWR frequency and associated CA1 neuronal activity were modulated by reward delivery under head-fixed conditions. To do this, LFPs were recorded from the CA1 region and aligned identified SWR events (Fig. 2*A*) to the timing of water-reward administration (Fig. 2*B*).

Water delivery produced a distinct change in ripple dynamics (Fig. 2*B*,*C*). Although some sessions showed a transient increase in SWR frequency immediately after reward delivery (Fig. 2*D*; *p* = 0.018 for 0–0.5 s; *p* = 1.52 × 10^−4^ for 0–2.5 s; Wilcoxon signed-rank test), the dominant response across the population was a prolonged suppression of SWRs following the initial response (Fig. 2*E*; *p* = 2.01 × 10^−5^; Wilcoxon signed-rank test for 0–2.5 s after reward; Table 1). To address the concern of pseudoreplication and confirm that the overall effect does not stem from a single outlier animal, we next fit a linear mixed-effects model (see Materials and Methods). The model yielded a significantly negative intercept (*β* = −0.775, *p* < 0.001), indicating that, on average, the overall suppression effect remains robust once we properly account for repeated measures across subjects. This pattern suggests that in a head-fixed context, the network's response to reward differs from the classic enhancement observed during consummatory behavior in freely moving animals.

**Table 1.** Statistical summary

Figure	Data	Rats	Primary statistic	Comparison	Statistic
2*D*	SWR rate (Hz)	*N* = 1 rat	Wilcoxon signed-rank test	Baseline vs 0–0.5 s window	*n* = 59 trials, *W* = 510, *p* = 0.018
2*D*	SWR rate (Hz)	*N* = 1 rat	Wilcoxon signed-rank test	Baseline vs 0–2.5 s window	*n* = 59 trials, *W* = 730, *p* = 1.52 × 10^−4^
2*E*	SWR rate (Hz)	*N* = 4 rats	Wilcoxon signed-rank test	Baseline vs 0–0.5 s window	*n* = 66 sessions, *W* = 213, *p* = 0.24
2*E*	SWR rate (Hz)	*N* = 4 rats	Wilcoxon signed-rank test	Baseline vs 0–2.5 s window	*n* = 66 sessions, *W* = 945, *p* = 2.01 × 10^−5^
3*A*	SWR rate (Hz)	*N* = 4 rats	Wilcoxon signed-rank test	Baseline vs 0–2.5 s window	*n* = 66 sessions, *W* = 809, *p* = 1.22 × 10^−3^
3*B*	SWR rate (Hz)	*N* = 4 rats	Wilcoxon signed-rank test	Baseline vs 0–2.5 s window	*n* = 66 sessions, *W* = 998, *p* = 4.78 × 10^−2^
3*C*	SWR rate (Hz)	*N* = 4 rats	Wilcoxon signed-rank test	Baseline vs 0–2.5 s window	*n* = 66 sessions, *W* = 845, *p* = 4.50 × 10^−2^
3*D*	SWR rate (Hz)	*N* = 4 rats	Wilcoxon signed-rank test	Baseline vs 0–2.5 s window	*n* = 66 sessions, *W* = 556, *p* = 4.83 × 10^−2^
3*E*	SWR rate (Hz)	*N* = 4 rats	Wilcoxon signed-rank test	Baseline vs 0–2.5 s window	*n* = 66 sessions, *W* = 735, *p* = 7.44 × 10^−3^
3*F*	SWR rate (Hz)	*N* = 4 rats	Wilcoxon signed-rank test	Baseline vs 0–2.5 s window	*n* = 66 sessions, *W* = 691, *p* = 3.22 × 10^−3^
3*G*	SWR rate (Hz)	*N* = 4 rats	Wilcoxon signed-rank test	Baseline vs 0–2.5 s window	*n* = 66 sessions, *W* = 905, *p* = 4.47 × 10^−5^
3*H*	SWR rate (Hz)	*N* = 4 rats	Wilcoxon signed-rank test	Baseline vs 0–2.5 s window	*n* = 66 sessions, *W* = 845, *p* = 4.50 × 10^−2^
4-1*A*	SWR rate (Hz)	*N* = 2 rat	Wilcoxon signed-rank test	Baseline vs 0–2.5 s window. Delta on epochs	ROI-1: *n* = 12 sessions, *W* = 34, *p* = 1.61 × 10^−4^; ROI-2: *n* = 12 sessions, *W* = 18, *p* = 0.0181; ROI-3: *n* = 12 sessions, *W* = 31, *p* = 0.0018; ROI-4: *n* = 12 sessions, *W* = 8, *p* = 0.0593; ROI-5: *n* = 12 sessions, *W* = 29, *p* = 1.18 × 10^−3^; ROI-6: *n* = 12 sessions, *W* = 46, *p* = 2.57 × 10^−9^; ROI-7: *n* = 12 sessions, *W* = 52, *p* = 1.71 × 10^−12^; ROI-8: *n* = 12 sessions, *W* = 48, *p* = 2.59 × 10^−10^
4-1*B*	SWR rate (Hz)	*N* = 2 rat	Wilcoxon signed-rank test	Baseline vs 0–2.5 s window. Delta off epochs	ROI-1: *n* = 12 sessions, *W* = 2, *p* = 0.56933; ROI-2: *n* = 12 sessions, *W* = 20, *p* = 0.0203; ROI-3: *n* = 12 sessions, *W* = 31, *p* = 0.00172; ROI-4: *n* = 12 sessions, *W* = 16, *p* = 0.0771; ROI-5: *n* = 12 sessions, *W* = 20, *p* = 0.02757; ROI-6: *n* = 12 sessions, *W* = 22, *p* = 0.00016; ROI-7: *n* = 12 sessions, *W* = 10, *p* = 0.0321; ROI-8: *n* = 12 sessions, *W* = 3, *p* = 0.1920

To validate our SWR detection approach and assess the functional relevance of these events, their relationship with neuronal firing was examined (Adaikkan et al., 2024). An increase in the multi-unit activity (MUA, 500–3,000 Hz band filtered signal) related to a high ripple-band power was observed (Fig. 2*A*). Additionally, using a subset of recordings (*n* = 12 sessions) where single-unit activity was simultaneously acquired, cross-correlograms were constructed between neuronal firing times and SWR peaks (Fig. 2*F*). This analysis revealed a significant increase in spike discharge coincident with SWR events, confirming that the detected ripples corresponded to genuine hippocampal network activity.

Finally, changes in spike firing rates of CA1 neurons were examined around reward delivery (Fig. 2*G*). Consistent with the notion that reward information can transiently enhance hippocampal excitability (Singer and Frank, 2009), most neurons showed increased firing shortly after reward. Nonetheless, a subset of neurons exhibited no significant change or even a reduction in firing, highlighting the heterogeneity of hippocampal network responses to reward events (Sakairi et al., 2025).

These results demonstrate that reward delivery under head-fixed conditions modulates hippocampal SWR activity, with a complex temporal pattern characterized by transient increases in some sessions but a more robust, long-lasting suppression observed across the population.

### Modulation of SWRs by orofacial and body movements

Next, the relationship between SWR occurrence and the onset of movement events associated with different body regions was investigated. To avoid confounding influences of reward-related effects on ripple activity, all movement events occurring within ±12 s of reward delivery were excluded. With this constraint in place, SWR activity was aligned to the onset of detected movements in specific ROIs, including the tongue, jaw, nose, whiskers (lateral and front), forelimbs, trunk, and tail. SWR frequencies in a baseline window preceding the movement event were compared with a test window immediately following it.

**Figure 3.**
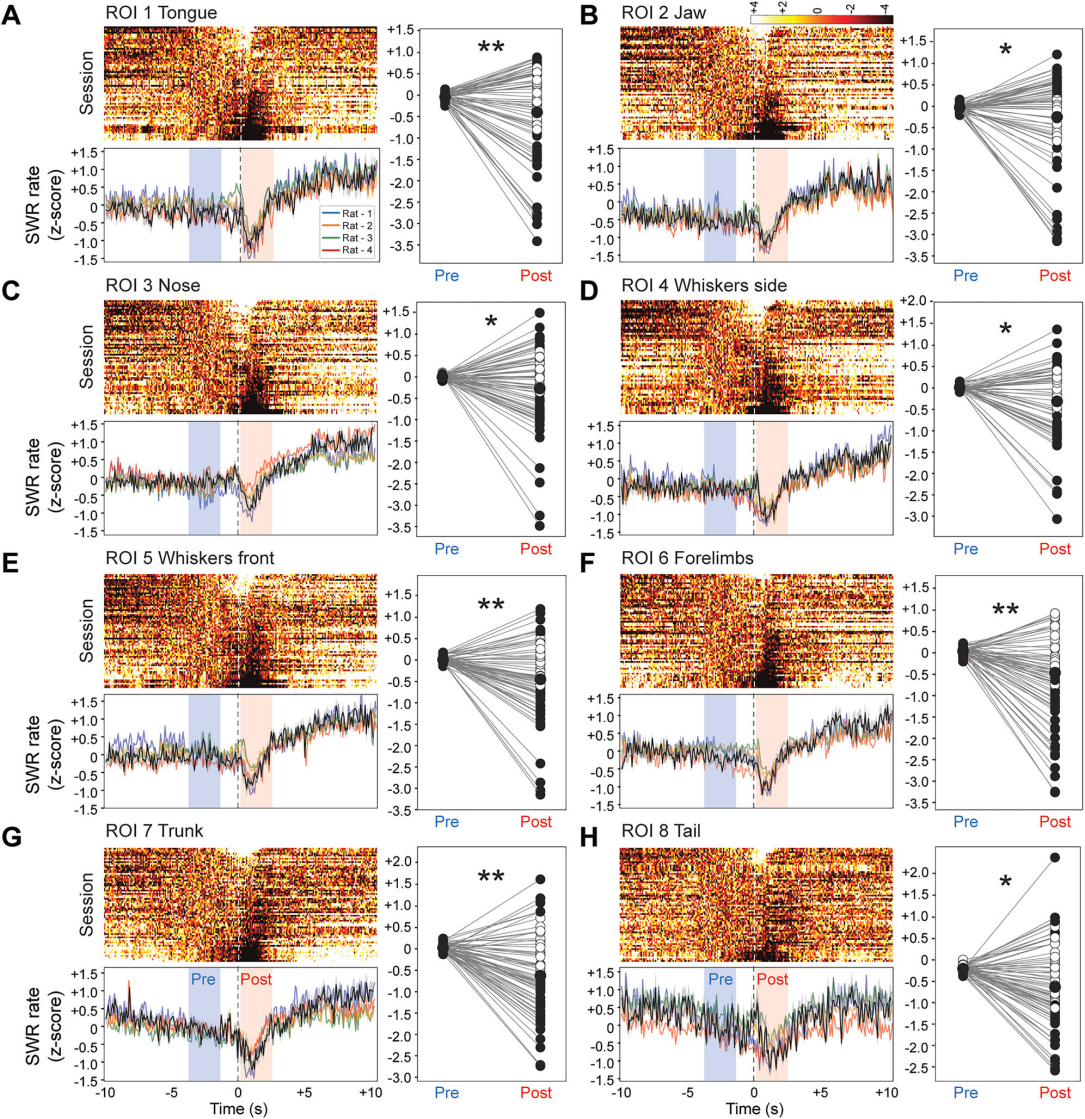
Modulation of SWR activity by orofacial and body movements, independent of reward proximity. ***A***, Top left, Population-level heat map of SWR activity aligned to the onset of tongue movements (top) and the corresponding PETH of SWR frequency (bottom). In the PETH plots, the population average is shown in gray with the SEM shaded, and each rat's average PETH is displayed in a different color. Blue shading indicates the baseline (Pre) window, and light red shading denotes the post-movement (Post) window. On the right of each panel, dot plots compare SWR activity between these two windows for each session; filled symbols indicate sessions with significant changes. ***B***, SWR activity aligned to jaw movements. ***C***, SWR activity aligned to nose movements. ***D***, SWR activity aligned to whisker lateral movements. ***E***, SWR activity aligned to whisker front movements. ***F***, SWR activity aligned to forelimb movements. ***G***, SWR activity aligned to trunk movements. ***H***, SWR activity aligned to tail movements (*p < 0.05; **p < 0.01; Wilcoxon signed-rank test).

Orofacial movements led to a predominant decrease in SWR frequency across sessions (Fig. 3*A–E*; Table 1). Although a minority of sessions showed increased or unchanged SWR activity, the overall trend was a significant reduction relative to baseline levels. This reduction was similarly observed for movements of other body parts, including forelimbs, trunk, and tail (Fig. 3*F–H*). Although session-to-session variability existed (with some sessions showing increases or no change), the dominant population-level pattern across all these ROIs was a reduction in SWR activity associated with movement events. In each case, the population PETHs revealed a downward shift in SWR frequency following the onset of the corresponding body part movement.

Together, these findings indicate that hippocampal SWRs are generally suppressed around the time of active orofacial and body movements, even when excluding movements that occur near reward delivery. This suggests that hippocampal network states conducive to SWR generation are less prominent when the animal engages in even minor, localized movements. This relationship was consistent across multiple ROIs, underscoring the robust link between behavioral activity and hippocampal ripple dynamics.

### Delta power correlates with SWR band activity and influences movement-related modulations

**Figure 4.**
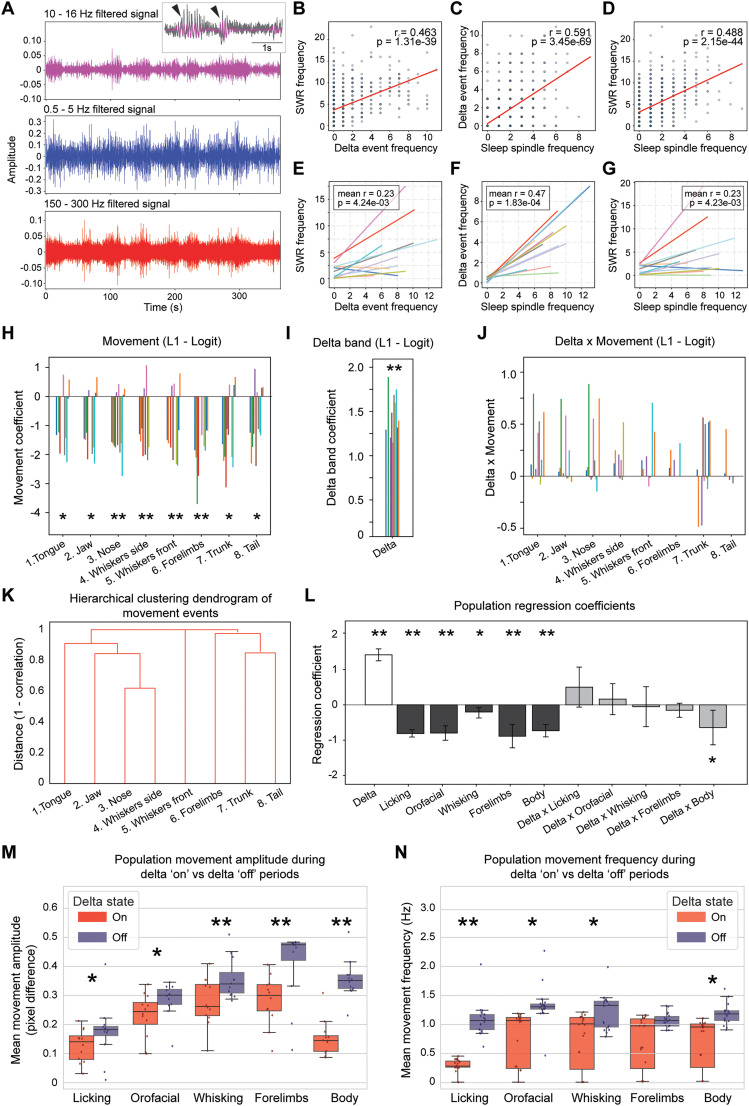
Delta-dependent gating of hippocampal sharp-wave ripples and dissociation from movement-related suppression. ***A***, Band-specific LFP traces. Representative 600 s excerpt of a single recording session showing 10–16 Hz filtered-band (magenta), delta-band (0.5–5 Hz, blue), and ripple-band (150–300 Hz, red) signals. The inset highlights detected spindle events (magenta, tick marks) over the raw LFP. ***B***, Delta events versus ripple frequency correlation. Ten-second binned scatterplot from the same session relating delta-event rate to SWR rate. Red line, Least-squares fit; Pearson’s *r* = 0.463, *p* = 1.31 × 10^−39^. ***C***, Spindle events versus delta events correlation. Scatterplot and linear fit (red) for spindle-event rate against delta-event rate (*r* = 0.591, *p* = 3.46 × 10^−69^). ***D***, Spindle events versus ripple frequency correlation. Relationship between spindle-event rate and SWR rate in the same session (*r* = 0.488, *p* = 2.15 × 10^−44^). ***E***, Population delta–ripple correlation. Lines represent individual sessions (*n* = 12); inset gives the mean Fisher-z-transformed *r* values (mean = 0.23) and one-sample *t* test versus 0 (*p* = 0.0042). ***F***, Population spindle–delta correlation. Same format as ***E***; mean *r* = 0.47, *p* = 0.0001. ***G***, Population spindle–ripple correlation. Same format as ***E***; mean *r* = 0.23, *p* = 0.0042. ***H***, Logistic-regression coefficients for individual ROI movements. L1-regularized logistic regression predicting SWR probability from single-ROI movement events. Error bars show coefficients from each session. ***I***, Logistic-regression coefficients for delta power. Corresponding positive coefficients for delta-band power across sessions. ***J***, Interaction coefficients (delta × movement). Interaction-term estimates for each ROI; most centered on zero, indicating little synergistic or antagonistic modulation beyond the main effects. ***K***, Hierarchical clustering of ROI movements. Dendrogram constructed from 1—correlation distances of movement time series, revealing clusters of correlated motor motifs (e.g., trunk–tail). ***L***, Logistic-regression coefficients after grouping ROIs. Coefficients recomputed with predictors collapsed into Licking, Orofacial, Whisking, Forelimbs, and Body movements categories. Delta remains significantly positive, grouped movements remain negative, and only the Delta × Body interaction reaches significance (*p* < 0.05). ***M***, Movement amplitude during delta states. Box-and-whisker plots comparing mean pixel-difference amplitude for five grouped motor categories during delta-on (red) versus delta-off (blue) epochs. Dots, individual sessions (Wilcoxon signed-rank). ***N***, Movement frequency during delta states. Mean event rate (Hz) for the same categories and statistical testing as in ***H***. Error bars, SEM across sessions; *p < 0.05; **p < 0.01. Please see Extended Data Figures 4-1, 4-2 for additional data.

To further explore the interplay between behavioral states and SWRs, it was examined how fluctuations in cortical LFP patterns, i.e., delta power (0.5–4 Hz) and sleep spindles, correlate with hippocampal ripple activity (150–250 Hz). Across the 12 sessions obtained from two rats, moment-by-moment fluctuations in the cortical delta envelope provided a reliable index of hippocampal ripple probability. Elevated delta power was accompanied by higher SWR rates (Fig. 4*A*,*B*,*E*). Delta peaks also coincided with sleep spindle events, which themselves correlated with ripples (Fig. 4*C*,*D*,*F*,*G*). These data position delta oscillations and sleep spindles (associated with low arousal and sleep pressure) as a broad, brain-state gate that raises the baseline excitability of the hippocampal network. Also, the regression between sleep spindles and delta events exhibited an intercept close to zero, implying that spindle events are minimal or nearly absent in the absence of delta events (Fig. 4*F*). In contrast, both delta–SWR (Fig. 4*E*) and spindle–SWR (Fig. 4*G*) correlations showed distinctly positive intercepts, indicating that SWRs occur at low but detectable rates even when spindle or delta events are scarce. This synchronous modulation aligns with previous studies linking large-scale brain states, such as sleep or quiescent wakefulness, to coordinated changes in hippocampal ripple generation (Sirota et al., 2003; Mölle et al., 2006; Goldstein et al., 2018).

10.1523/ENEURO.0012-25.2025.f4-1 Figure 4-1 Impact of cortical delta power on movement-related hippocampal SWR modulation across behavioral states. (A) Movement-related modulation of SWRs during delta-on (sleep-like) epochs. For each ROI (tongue, jaw, nose, whiskers lateral, whiskers front, forelimbs, trunk, tail), the left panels show the average PETHs of SWR frequency aligned to movement events. Blue and red shaded areas represent the baseline and test windows, respectively. The right panels illustrate session-by-session comparisons of pre- vs. post-movement SWR activity (blue and red dots, respectively), with filled symbols indicating sessions that reached statistical significance. (B) The same analysis as in (A), but during delta-off (quiet awake) epochs. (* = p < 0.05; ** = p < 0.01; Wilcoxon signed-rank test). Download Figure 4-1, TIF file.

10.1523/ENEURO.0012-25.2025.f4-2 Figure 4-2 (A) Session-by-session matrix illustrating changes in SWR frequency aligned to the onset of movements for multiple body parts during delta-off epochs (tongue, jaw, nose, whiskers side, whiskers front, forelimbs, trunk, tail). Each row represents one recording session, and each column corresponds to a specific body part. Colors indicate the normalized change in SWR activity relative to baseline: blue denotes a decrease, and red denotes an increase. Statistical significance, assessed by Wilcoxon signed-rank test, is indicated by asterisks (*p < 0.05; **p < 0.01; Wilcoxon signed-rank test). (B) Same as (A) for movements for multiple body parts during delta-on epochs. Download Figure 4-2, TIF file.

We next asked whether self-generated behavior could override this permissive influence. L1-regularized logistic models that treated each video-derived motor feature, delta events, and their interaction as simultaneous predictors revealed a consistent pattern: every movement carried a negative weight whereas delta remained strongly positive (Fig. 4*H*,*I*). Interaction terms were near zero, indicating that movement suppression and delta facilitation add linearly rather than synergistically (Fig. 4*J*). Separating perimovement analyses by brain state revealed an identical qualitative outcome. In putative sleep-like (delta-on) epochs, ripple rates dropped the moment a movement began; the same pattern was evident, albeit slightly weaker, during delta-off quiet wakefulness (Table 1; Extended Data Fig. 4-1). Taken together, these findings demonstrate that (1) cortical delta oscillations promote hippocampal ripples and (2) the suppressive influence of movement on ripples is remarkably robust, though the degree of modulation sometimes varied across sessions, surviving even within delta-dominated, putative sleep-like periods. While the overarching pattern of movement-induced SWR suppression remained consistent across delta-on and delta-off conditions (Table 1; Extended Data Fig. 4-2), subtle differences in the magnitude or significance of SWR modulation suggest that the underlying brain state, as reflected by delta power, can influence the extent to which hippocampal ripples are affected by behavioral events.

Because several ROIs captured partially overlapping behaviors, we next examined their redundancy. Hierarchical clustering of the eight-movement time series based on 1 − *R* revealed two principal clusters: (1) tongue, jaw, nose, and whisker motions (orofacial) and (2) trunk and tail displacements accompanied by forelimb retractions (body-centered; Fig. 4*K*). Highly correlated ROIs were further consolidated into five broad categories (Licking, Orofacial, Whisking, Forelimbs, Body). Refitting the model with these five movement groups produced similar results (Fig. 4*L*): delta remained a strong positive predictor (*p* < 0.01), all grouped movements were negative (*p* < 0.01 for four of the five), and only the delta × Body interaction attained significance (*p* = 0.03), indicating a mildly weaker suppression of ripples by gross body motions during high-delta periods. Although delta events coincided with visibly quieter behavior, animals were not completely still. Both the amplitude and the frequency of detected movements fell during delta-on windows, yet small movements persisted (Fig. 4*M*,*N*). These combined behavioral and electrophysiological markers showed that the animal can exhibit movements and enter a sleep-like state despite being head fixed (Movies 2, 3; Tiriac et al., 2012; Dos Santos et al., 2019).

**Movie 2.** Brief adjustments. Twenty-four-second recording aligned to water-reward delivery (time 0 s). Layout is identical to Movie 1. Camera views capture short forelimb and orofacial movements. Corresponding transient peaks appear in the forelimb and orofacial ROI traces, whereas other ROIs remain near baseline. Ripple-band LFP is displayed continuously. [View online]

**Movie 3.** Prolonged immobility. Twenty-four-second recording aligned to water-reward delivery (time 0 s). Layout is identical to Movie 1. Camera views show the rat remaining largely motionless for the duration of the clip. All ROI traces remain at baseline except for occasional minor deflections. The hippocampal 150–250 Hz LFP is displayed in the bottom left panel. [View online]

**Figure 5.**
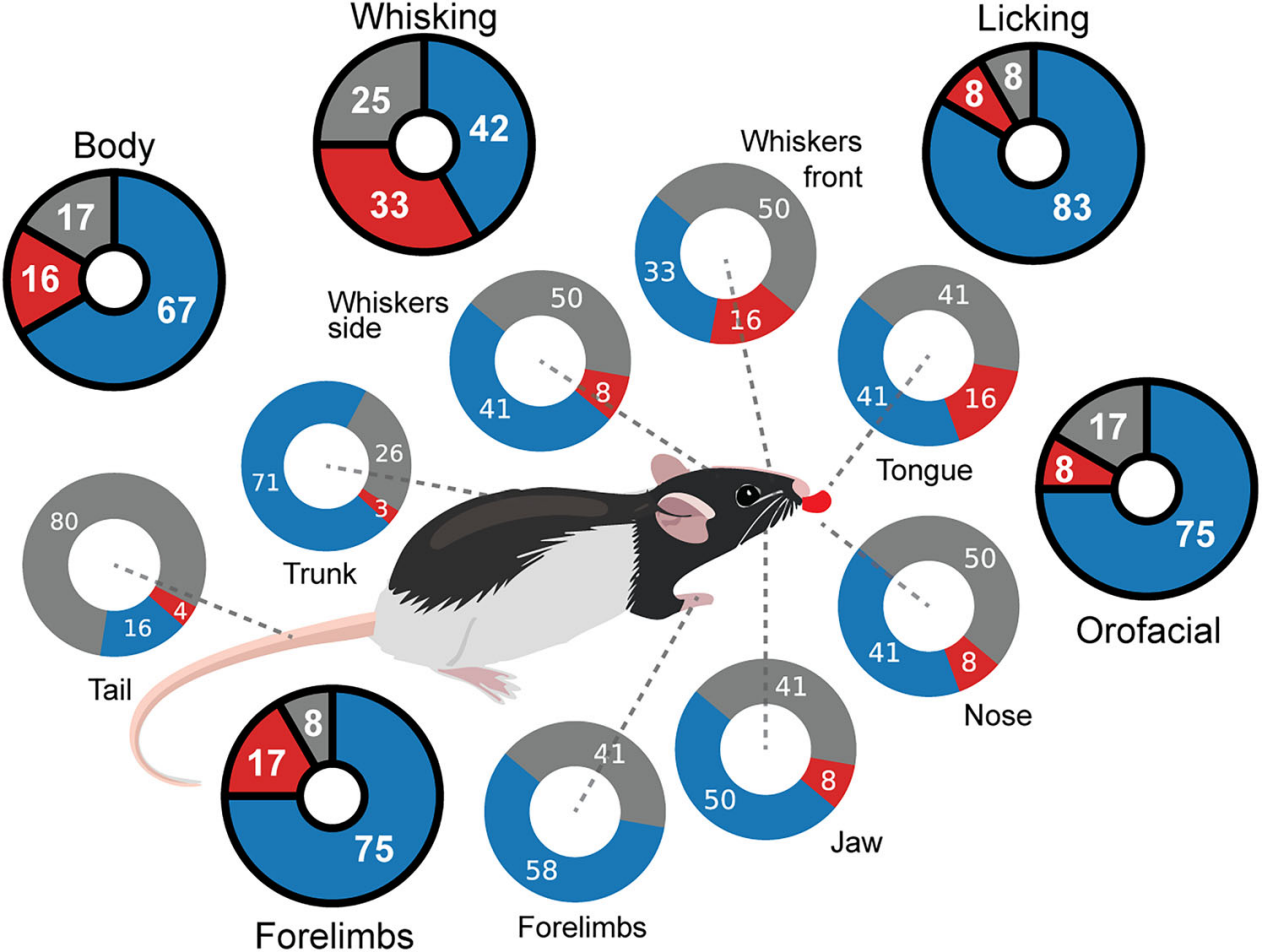
Summary of session-wise impact of individual and grouped movements on ripple likelihood. Schematic summary of the SWR activity related to body movements. Each body part of the rat is paired with a donut plot indicating the percentage of sessions that showed significant decreases (blue), significant increases (red), or no significant change (gray) in SWR activity. The outer donuts summarize the same analysis after pooling ROIs into five movement clusters (Licking, Whisking, Orofacial, Forelimbs, and Body). Colors and numeric labels follow the same convention as the inner rings.

In summary, these results demonstrate that SWR activity is tightly coupled to cortical delta oscillations and that this relationship remains evident when examining the effects of movement on SWRs across different brain states. Whether during delta-on (sleep-like) or delta-off (awake quiet) epochs, movement-related suppression of SWRs persists, being consistent across body parts (Fig. 5), underscoring the multifaceted and state-dependent regulation of hippocampal network dynamics.

## Discussion

In this study, we investigated how hippocampal SWRs are modulated by behavioral states, reward delivery, and localized movements in head-fixed rats. Building on previous work that established SWRs as critical for memory consolidation, decision-making, and planning (Ji and Wilson, 2007; Girardeau and Zugaro, 2011; Buzsáki, 2015) and that their disruption can lead to pathological outcomes (Suh et al., 2013; Zhen et al., 2021), our findings provide new insights into the dynamic interplay between hippocampal network activity and specific behavioral components.

Traditionally, research conducted in freely moving rodents has outlined a clear dichotomy between preparatory and consummatory behavioral states (O'Keefe and Nadel, 1978; Csicsvari et al., 2007; Buzsáki, 2015). During preparatory periods, when animals actively pursue rewards, hippocampal activity is dominated by theta oscillations, which are associated with spatial processing and attention. In contrast, when animals transition into a consummatory phase (such as consuming a reward), SWRs typically increase, suggesting the facilitation of the consolidation and replay of recently acquired information (Singer and Frank, 2009; Buzsáki, 2015). These distinctions have been observed in tasks where animals are engaged in foraging, navigation, and consumption.

Our findings, however, suggest that under head-fixed conditions, the modulation of SWRs by reward delivery is more nuanced and context dependent. Although some sessions exhibited a brief, transient increase in SWR frequency immediately after reward delivery (Fig. 2), the predominant pattern was a prolonged suppression of SWRs across the population (Fig. 5). This suppression was observed across a range of movements, including even minimal orofacial and body adjustments, supporting the idea that any motor activity can inhibit hippocampal ripple generation. In a similar direction, during delta-off epochs, regardless of whether some sessions and ROIs display no significant changes or occasionally an increase in SWRs, the dominant pattern remains a suppression of SWR activity around movement events. This finding is consistent with recent observations reporting similar results in head-fixed rodents (Klee et al., 2021, Sakairi et al., 2025). Thus, although hippocampal networks retain the capacity for SWR enhancement under certain conditions, head fixation and the associated constraints on naturalistic movement and behavioral sequences appear to reshape the interplay between reward consumption and ripple generation. The lack of full-bodied locomotion and extensive exploratory behaviors in head-fixed paradigms indicates that in our experimental setup the behavioral state is not directly analogous to the preparatory and consummatory phases seen in freely moving tasks.

Beyond reward modulation, we found that even minor orofacial and body movements strongly suppressed SWR occurrence (Figs. 3, 4). This phenomenon extends the classic view that SWRs occur predominantly during immobility, quiet wakefulness, and sleep (Foster and Wilson, 2006; Buzsáki, 2015). These results reveal that small, localized movements, far from the vigorous locomotion typically studied, are sufficient to disrupt the conditions favoring SWR generation. While in freely moving rodents, preparatory states often involve exploratory locomotion that can suppress ripples (Sullivan et al., 2011; Kay and Frank, 2019), our findings suggest that even in a head-fixed setting, minimal motor engagement still reallocates hippocampal resources away from internal replay and toward processing immediate sensory-motor inputs. Thus, the suppression of SWRs during small movements may represent a fundamental and conserved principle of hippocampal function, one that transcends differences in the environmental complexity or degree of physical freedom available to the animal. A potential mechanism underlying this movement-induced suppression of SWRs is the cholinergic system, particularly inputs from the medial septum. Previous work has shown that optogenetically stimulating septal cholinergic neurons both enhances theta oscillations and suppresses sharp-wave ripples (Vandecasteele et al., 2014) and that in natural conditions, acetylcholine (ACh) levels rise during active behaviors and fall during ripple generation (Zhang et al., 2021). Because cholinergic neuron firing correlates with movement, it is plausible that even small motor activity boosts ACh release, thereby inhibiting the hippocampal network states that support SWR occurrence. Future experiments could test this hypothesis directly.

The analyses also showed that SWR activity is closely coupled with cortical delta oscillations (Fig. 4), a known hallmark of global brain states such as sleep and quiet wakefulness (Sirota et al., 2003; Mölle et al., 2006). Interestingly, spindle events appeared fundamentally dependent on cortical delta states, as evidenced by the near-zero intercept of their correlation (Fig. 4*F*). In contrast, both delta–SWR and spindle–SWR correlations had distinctly positive intercepts (Fig. 4*E*,*G*), suggesting that hippocampal ripples, although strongly facilitated by cortical delta and spindle activity, can nevertheless occur spontaneously, at baseline levels, even when such cortical signals are sparse or absent. This hierarchical organization implies that SWRs represent an intrinsic hippocampal phenomenon, whose expression is modulated, but not exclusively determined, by cortical state fluctuations. Even during high delta and high sleep spindles epochs, putative sleep, rodents are not perfectly still. REM bouts contain rapid limb and whisker twitches, whereas non-REM periods feature brief micro-arousals with small head or body adjustments that momentarily activate forebrain sensorimotor networks (Tiriac et al., 2012; Dos Santos et al., 2019). Whether in these delta-on (sleep-like) or delta-off (quiet-awake) epochs, the fundamental movement-related suppression of SWRs persisted (Fig. 4, Extended Data Fig. 4-1). Thus, while delta power and spindle activity enhance the excitability landscape promoting SWR generation, they do not override movement-related suppression. This interplay between global brain-state signals and local motor-related inputs allows the hippocampus to flexibly balance mnemonic processing with the demands imposed by ongoing behavioral interactions with the environment (Jadhav et al., 2012; Ólafsdóttir et al., 2018).

These results further underscore the complexity of SWR regulation: reward and consummation, in which freely moving studies have often associated with enhanced SWR activity (Singer and Frank, 2009), here yielded a more context-sensitive pattern. The restricted and highly controlled conditions of head fixation likely reduce spatial navigational demands and the variety of preparatory actions that characteristically precede reward consumption in free-ranging foraging scenarios. As a result, hippocampal circuits may shift away from replaying reward-related experiences and instead maintain a lower excitability state after a brief postreward transient. This difference highlights the importance of behavioral context and environmental constraints in interpreting hippocampal activity patterns and their functional significance.

Though our head-fixed preparation allowed precise monitoring of small movements and provided stable electrophysiological recordings, it also limited the behavioral repertoire and spatial exploration of the animals. Future research should replicate these experiments in freely moving settings to confirm the generality of our findings, particularly regarding the dissociation between preparatory and consummatory states (e.g., under more cognitive demanding tasks, requiring a higher engagement in attention, discrimination, or decision-making). Additionally, exploring how SWR modulation by movement and reward differs across hippocampal subregions or species could clarify the universality of these regulatory mechanisms.

In summary, this study provides an alternative view of SWRs as dynamic phenomena influenced not only by global brain states and reward conditions but also by small, localized movements (Fig. 5). By comparing our results with the classical paradigms of freely moving animals, we highlight that SWR modulation is context dependent. This contributes to a more nuanced understanding of hippocampal function, demonstrating that even within similar behavioral states (e.g., consummatory behavior), the neural underpinnings of SWR generation can shift depending on the sensory-motor context and environmental constraints. Such flexibility ensures that the hippocampus can continuously adapt its memory-related activity amid an ever-changing environment.

## Synthesis

Reviewing Editor: Mark Sheffield, University of Chicago Division of the Biological Sciences Decisions are customarily a result of the Reviewing Editor and the peer reviewers coming together and discussing their recommendations until a consensus is reached. When revisions are invited, a fact-based synthesis statement explaining their decision and outlining what is needed to prepare a revision will be listed below. The following reviewer(s) agreed to reveal their identity: NONE.

Both reviewers find the study to be addressing an important and underexplored question with potential value to the field. However, several key concerns must be addressed to ensure the findings are statistically sound, methodologically clear, and effectively communicated.

A primary concern is the statistical analysis framework, as the current approach does not account for repeated measures within individual animals. Both reviewers strongly recommend implementing a mixed-effects model or, at minimum, breaking down effects by animal to ensure robust conclusions. Additionally, clearer characterization of movements is needed, including clustering movement types and quantifying their frequency and variability across sessions.

Further clarification is also required regarding the head-fixation procedure, potential stress effects, and the age of the animals. Several methodological inconsistencies need to be resolved, including session sorting in figures, delta activity definitions, and reference accuracy. Improved figure clarity and organization will further enhance the manuscript.

During the consultation, both reviewers emphasized that while the study presents an interesting finding, further statistical and methodological refinements are necessary. The detailed reviewer comments that follow provide specific guidance for these revisions.

Reviewer 1

This is a potentially interesting paper with the main claim being that sharp wave-ripples are suppressed by movements in head-fixed, non-locomoting rats. However, there are some points that require clarification to strengthen the evidence for this claim and to increase the impact of the work for the broad audience of eNeuro.

1. The results in Figure 2E, Figure 3, are not entirely convincing. Also, if I am understanding correctly, these are not two independent sets of samples, as the statistical test used assumes. Did the authors try to perform a repeated measures test with different sharp wave-ripple rates nested within individual animals? It is also questionable whether the Figure 4 results are meaningful from 1-2 rats, given that the individual measures are treated as independent samples, rather than repeated samples within subjects.

2. How were the rats trained to be head-fixed? This is unusual and seems like a stressful procedure for rats. Potential effects of stress on these results should be discussed.

3. There is a prior literature showing that ACh suppresses sharp wave-ripples, and cholinergic neurons, including in the medial septum, have been shown to increase their firing rates with movement. To increase the broad impact of these observations, the authors may want to discuss this work to suggest a potential mechanism underlying the observed effects.

Reviewer 2:

The authors describe a set of experiments involving acute recordings from the hippocampus of head-fixed rats. The rats were water restricted and received random water rewards while head-fixed. The authors report their findings of how sharp wave ripples (SWRs), an LFP event that has been tied to learning and memory reactivation, are related to reward delivery as well as orofacial and postural movements of the animals.

They find that rate of SWR is reduced following reward delivery. This is in contrast to the dominant finding that has been reported in the freely moving literature: that SWR increase following reward delivery. The authors use video frame analysis to find that the drop in SWR rate is accompanied by orofacial and postural movements during reward consumption, along with a small immediate increase around the actual reward delivery. The authors then go on to follow up this finding by looking at periods of spontaneous movement without reward, and finding that, on average, the SWR rate decreases, although this effect seems more variable. In the final section the authors describe an increase in SWR during increased delta band activity, which is in accord with the literature. They report some varying effects of the delta on/off state interacts with how movement modulates SWR.

I found the manuscript well organized and fairly straight-forward to follow. While I believe this work is of value to the community and is suitable for publication in a venue such as eneuro, I think that the manuscript in its current form requires significant improvement.

There were a quite a number of mistakes (missing references, references to data in figures that was not there) in the manuscript that reflect that it could use a bit more careful reading and editing.

Most importantly perhaps

-the overall effect needs to be more rigorously demonstrated across multiple animals / experience.

-the type of movement the animals are doing and how frequently needs to be better communicated.

I think the authors have a relevant finding, and if it holds up, this will make a nice publication.

Questions/Concerns about head-fixation

I have some question about the use of head-fixation in rats. There are a scant few rat head fixation studies in the literature, since naïve rats do not seem to tolerate it very well (compared to mice). The authors report that the rats were "comfortably head-fixed", I'm not really sure what this means. The authors go on to report body movements in the manuscript, but It is very hard to get a sense of how large these movements are and how frequent? Would they be considered struggling? Do the movements decrease over the session or over the sessions of training? Is there a lot of movement initially in the first hour as the electrodes are settling and then they are resigned to being head-fixed after that? I think many readers will have similar questions. Perhaps the easiest way to address this would be to include some supplementary video of the animal. Another suggestion would be to show an example session (in entirety) with the movement (and immobility periods) shown (figure 1 would be a good place for this..

Looking at the methods, I note that the weights would indicate that young animals were used in this experiment. Please include the ages in the methods, and include this as a caveat in the discussion if the animals used were juveniles.

Effects across animals.

Throughout the manuscript, the 66 sessions are mostly treated as (independent) observations, this would perhaps be ok if the effects were very strong or consistent. However, for the results describing the relationship of SWR rate to orofacial and body movements and the small rise after reward, the effects seem rather inconsistent. Many sessions clearly increase following movement onset figure 3. Indeed, this is acknowledged in the discussion section as "complex and context-dependent modulation of SWRs". I think without a clear grasp on what the context is, the authors cannot call it context-dependent modulation. At this point it is unexplained variation.

On possibility is that there is just one animal that is driving the effects they see. I would like the authors to consider the effects they report as a function of the animal ID, this might clarify the complex patterns they see. The hope is that the main effects would be seen across all animals.

Another potential factor could be how experienced each animal is with the overall task? All the sessions from the very start of training are included: perhaps there is a learning effect that explains the differential modulation as animals become more familiar with the setup? This would actually be an interesting effect. For instance, initially the rewards are entirely unexpected, but over experience animals will come to expect them (even if not their exact arrival times).

Another question I have is related to this is how are the 66 sessions sorted in the plots? There often seems to be some interesting gradient eg 2C top plots show short time scale SWR with reward for some sessions &2G shows the same effect but for different neurons. Which sessions are the neurons from? Are those with strong responses to reward from sessions that also show reward SWR modulation? Please describe how the individual sessions or neurons are sorted, and if the sorting is consistent across subpanels (eg figure 3).

.

A more appropriate statistical framework for analyzing the data might be to use a mixed effects model, where the hierarchical structure of the dataset is captured. I would refer the authors to the following article https://bmcneurosci.biomedcentral.com/articles/10.1186/1471-2202-11-5

Which explains how things can go wrong, and why mixed effects models are one solution.

As an absolute bare minimum, I would like to see any effects that you are claiming to be broken out by animal.

A more sophisticated statistical framework would also allow the authors to better answer the question posed by the delta state condition, which I believe is does movement modulation of SWR depend on Delta state? For this analysis I would suggest a multiple regression, that explicitly tests if there is 1) a main effect of delta state 2) an interaction of delta state and movement.

All the analysis would also be made much simpler by better characterization of the movements the author observes (see next point).

Better description of movement.

Throughout the manuscript, the 8 ROIs are treated as almost entirely separate measures. Presumably there is some correlation between them? Such a correlation could correspond to distinct behavioral motifs? The data that are presented in this manuscript are the raw features of movement, and as such I don't think are that useful to consider as separate entities in their own right. I would strongly advise the authors try to do some clustering on these features to discover If there are different movement categories, and then use this to analyze the neural data. The authors already describe two types of movement (orofacial reward related and trunk movements), so it would be nice to show some clustering of the video data features to back this up. This more compact description of the data categories could then be used in all later analysis and figures?

The authors describe movements as "subtle", I would recommend that this term is avoided, as it implies the movements are difficult to detect. Is this what they author mean? I assume the authors actually mean small movements. This brings me to a related point, that it is difficult for the reader to ascertain what types of movements we are taking about here, especially since the units are in this abstract pixel-wise distance. As well as a better characterization mentioned above, the authors could provide some sequences of video frames showing the actual movements along with the measures. Alternatively (and preferably) the authors should include some exemplar videos of typical movements so that the readers have a better idea of what is going on.

I also didn't get any idea of how frequent movements were. The authors state that "Quantification of these states (n = 66 sessions, 4 rats) demonstrated consistent transitions between these behavioral epochs across trials" What / where is the report of this quantification? What exactly was consistent? The fine timescale of the movement features is shown in figure 1E, but it would be great to have the larger time scale structure and some statistics (eg animals had orofacial movement bouts at a session frequency of ... Hz).

Delta section

I found this section to be the weakest and most confusing of the results. As the authors acknowledge, these effects are variable. Perhaps the effects will become clearer once broken out in the ways suggested above (by animal, by experience). It is also not entirely clear whether animals should be moving during nrem sleep? I would expect whisking and extremities twitching during REM but I would have expected very little movement during nrem sleep.

Discussion

I found the discussion section of the manuscript to be do a decent job at summarizing the study and covering interpretations. However, I thought that perhaps it was overselling the difference between the freely moving condition. For instance, it seems entirely consistent that SWR are inhibited by movement in general, whether by locomotion or the smaller postural and orofacial movements reported in this study. For freely moving experiments SWR rate increases when the animal stops to consume reward, whereas in this study it goes down as the animal moves from immobility to movement to collect the reward. I think the authors do make this point, but they do so in a rather round-about way.

The authors should also discuss that their task had no real operant condition, ie the animals were just randomly getting reward, whereas in most spatial tasks there is (as they mention) some navigational component.

I would also suggest, from a stylistic point of view, to be a little more economical with the poetic turns of phrase in the discussion: there are only so many tapestries or transcendences that the average reader can appreciate.

General points

In general, the manuscript requires some effort to improve the figures and text. For instance, some paragraphs in the body of the text read like figure legends, and just describe the details of the figure.

In regards to the figures, the approach taken has been to simply duplicate plots for the separate ROIs and make the reader do the work to try and digest a large amount of fairly raw data. I think this approach would be appropriate for a supplementary figure, but I would challenge the authors to present a more digested view of the data in the main figures. Additionally, the figure text, especially axis values, were in general too small.

Specific points

"periods of delta-on activity were defined as

those exceeding 4 standard deviations for at least 200 ms (Girardeau, 2021)" - this citation for the delta-on period Is a review, and I could not find any mention in there of these specific criteria in it... please refer to primary literature so readers can properly evaluate your detection criteria,

I find it anyway rather surprising that animals are falling asleep during head fixation. is there anything else that can confirm this as a sleep state (eg eye closure?)

At least one reference cited in the text but not in the bibliography

Klee, 2023

Please double check all references In the text are in the bibliogtarphy

In the methods the reward was at "random intervals (180 {plus minus} 40 s)," this is slightly unclear, do you mean random from a uniform distribution [140 - 220s]?

Spiking date are referenced in the main text "An increase in the

Multi-unit activity (MAU) related to a high ripple band power was observed (Figure 1A)" but data is not shown, fig 1A Is a diagram.

Multi-unit activity (MAU) < typo

Figure 1

The video images show a hand grip, which is not indicated in panel A.

Panel B has no time axis.

It would also be nice to show the ethogram (state and rewards) of the whole session, with the zoomed in section being the one shown currently.

D has no units

Figure 2G - no units

"Delta Power Correlates ...

Extending this analysis across all recorded sessions (n = 12), "

I could not work out where this n of 12 comes from. It is a subset of the all the sessions? But I couldn't find the criteria.

Figure 4 - the red lines are regressions, but the significance is a correlation? Why not use the p value from the regression?

Figure 5 is not mentioned in the results section. It seems to include results from the delta analysis and summary from the SWR movement section earlier on. I would suggest integrating these results into the respective figures as they come up in the results.

## Author Response

Dear Editor, Thank you for your thorough review and constructive feedback on our manuscript. We appreciate the careful evaluation and the opportunity to address the minor revisions requested. Below is our detailed response to each of your comments:

1. References and Approval Committees: − All references previously omitted due to the blind review process have now been included. − The names of the approval committees have been explicitly incorporated into the manuscript. − The citation originally listed as Sakairi (2024) (BioRxiv preprint) has been updated to Sakairi (2025),after its current peer-reviewed publication.

2. References on Head Fixation Procedures: − As suggested, references mentioned in our previous rebuttal letter concerning head fixation procedures have been integrated into the methodology section.

3. Clarification of Movement Descriptions: − All remaining instances of the term "subtle" (lines 493 and 457) have been revised to "small" to eliminate ambiguity and clearly convey our intended meaning.

4. Formatting Corrections: − Figure 4: The previously misformatted PDF conversion issue has been corrected. We have ensured the accurate upload of the TIF-format figure and verified proper formatting upon PDF conversion. − Table: The table has been revised, reformatted, and labeled. − Ticks: Ticks have been correctly added to Figure 4 panel A.

We believe these modifications fully address the editorial concerns. Thank you again for your valuable feedback and support throughout the review process.

We look forward to your positive consideration for publication.

Best regards, Alain Ríos, PhD.

Institute of Science Tokyo
