# Supplementary figures and images for: Modulation of Hippocampal Sharp-Wave Ripples by Behavioral States and Body Movements in Head-Fixed Rodents
Source: eNeuro. 2025 Jul 23;12(7):ENEURO.0012-25.2025. doi: 10.1523/ENEURO.0012-25.2025 (PMC12303588; doi:10.1523/ENEURO.0012-25.2025)

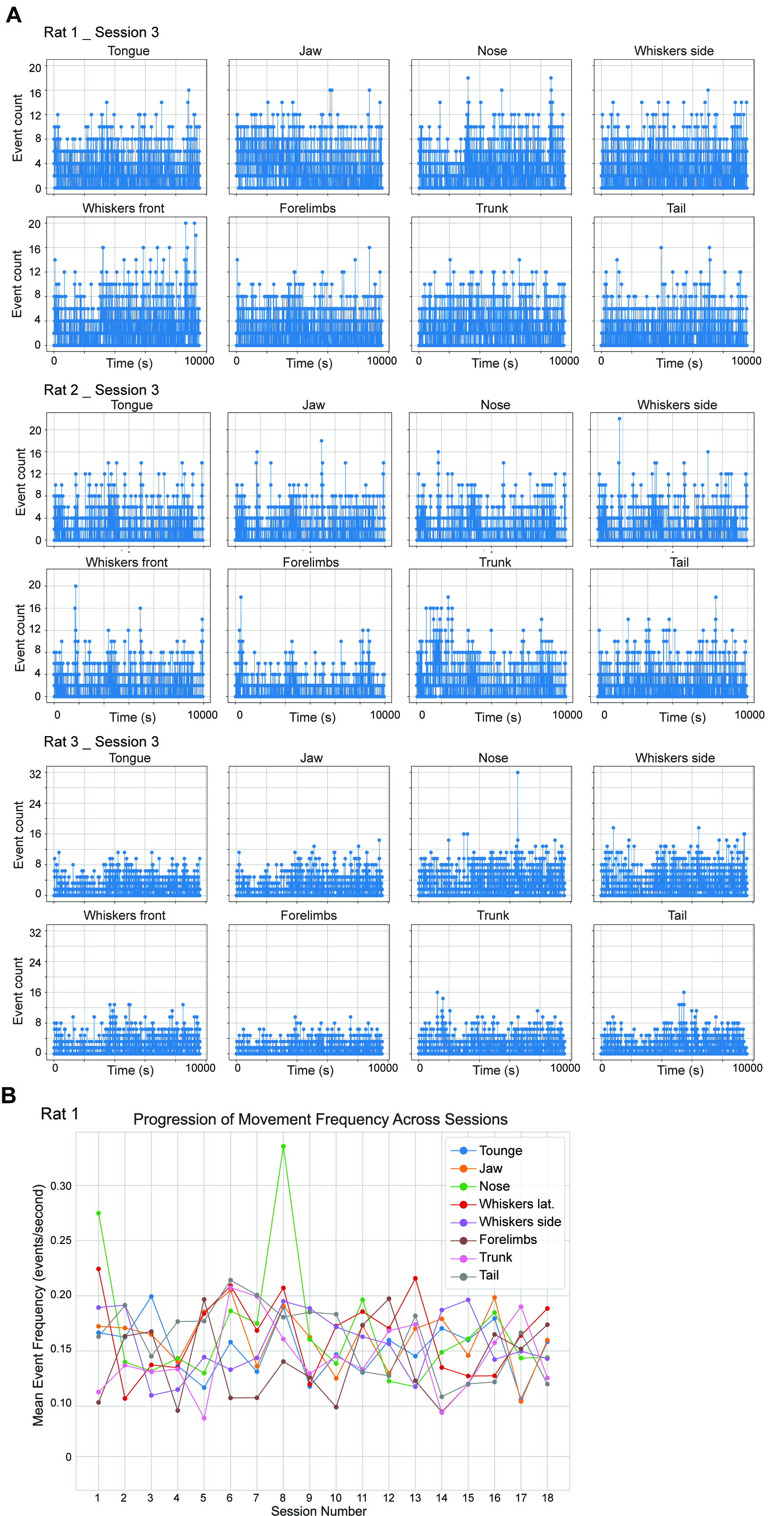

Supplement: Figure 1-1 — Within- and across-session progression of movement events. (A) Representative data from three rats illustrating the time course of movement events for each region of interest (ROI) within a single session. Each panel shows the event count over time for one ROI, allowing visualization of how movement frequency changes throughout the session. (B) Example progression of average movement event frequency (events/s) across multiple recording sessions for a single rat. Each trace corresponds to one ROI, revealing the relative stability or variability of movement patterns as the sessions progress. Download Figure 1-1, TIF file. [file eneuro-12-ENEURO.0012-25.2025-s002.tif]

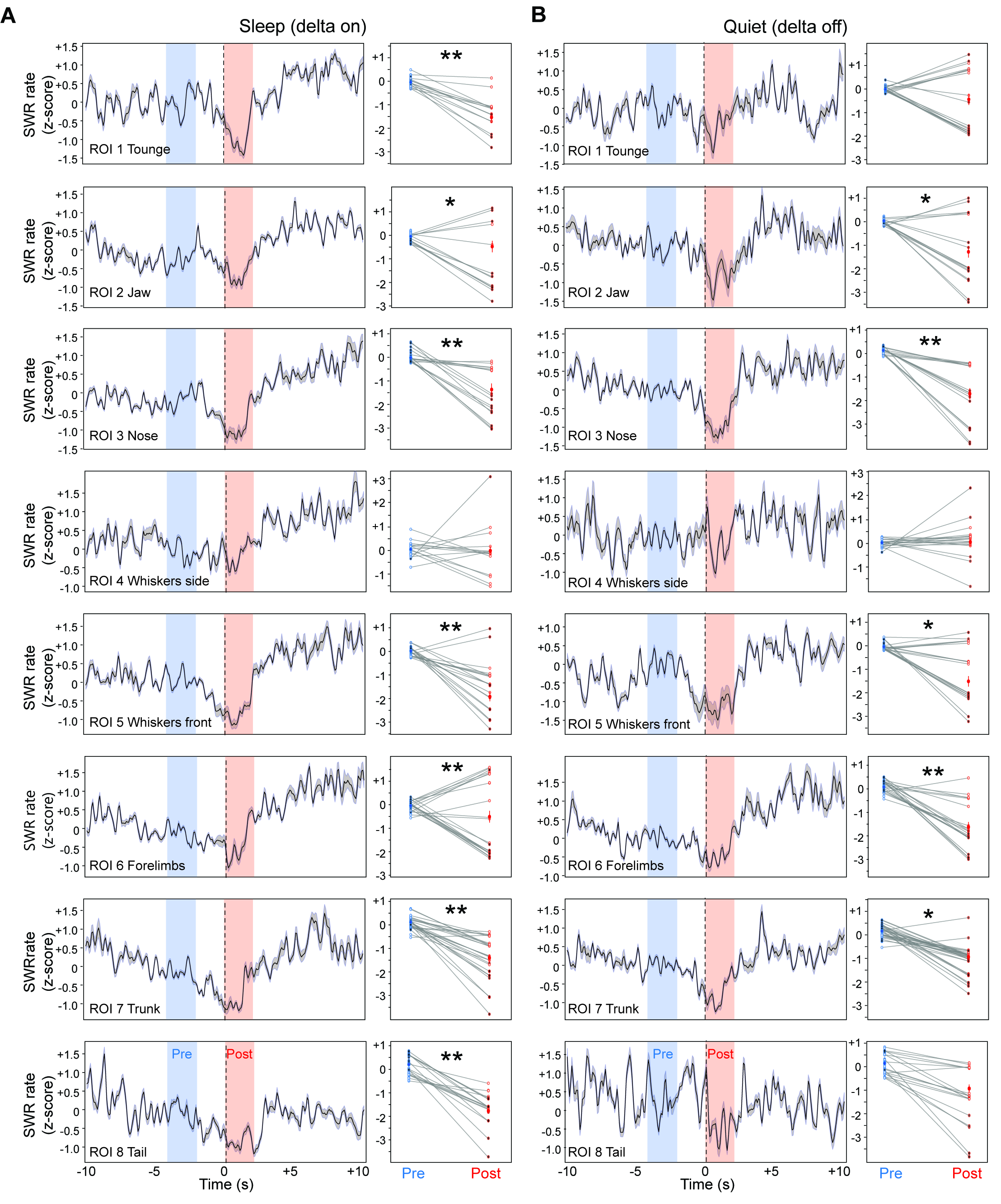

Supplement: Figure 4-1 — Impact of cortical delta power on movement-related hippocampal SWR modulation across behavioral states. (A) Movement-related modulation of SWRs during delta-on (sleep-like) epochs. For each ROI (tongue, jaw, nose, whiskers lateral, whiskers front, forelimbs, trunk, tail), the left panels show the average PETHs of SWR frequency aligned to movement events. Blue and red shaded areas represent the baseline and test windows, respectively. The right panels illustrate session-by-session comparisons of pre- vs. post-movement SWR activity (blue and red dots, respectively), with filled symbols indicating sessions that reached statistical significance. (B) The same analysis as in (A), but during delta-off (quiet awake) epochs. (* = p < 0.05; ** = p < 0.01; Wilcoxon signed-rank test). Download Figure 4-1, TIF file. [file eneuro-12-ENEURO.0012-25.2025-s003.tif]

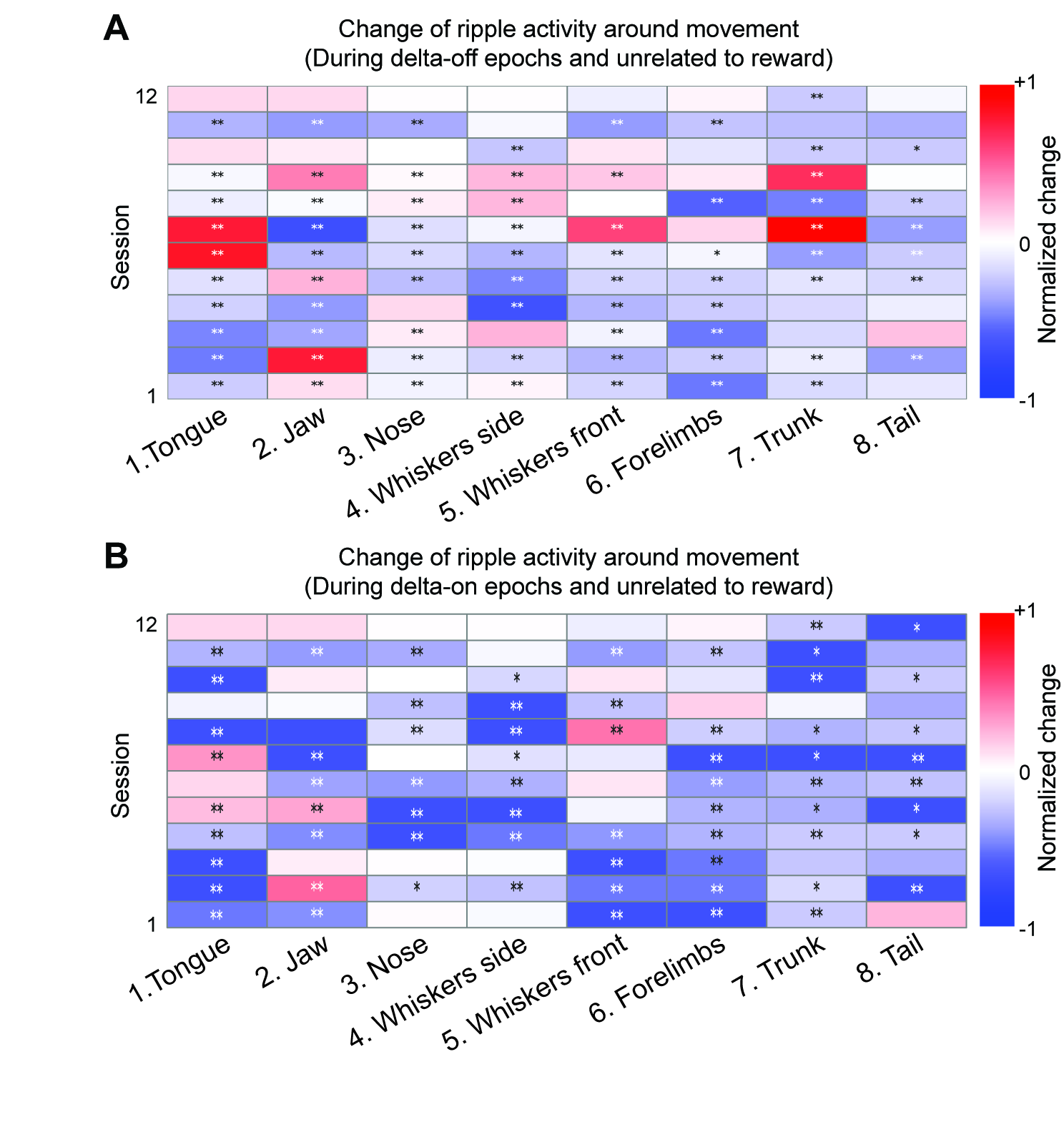

Supplement: Figure 4-2 — (A) Session-by-session matrix illustrating changes in SWR frequency aligned to the onset of movements for multiple body parts during delta-off epochs (tongue, jaw, nose, whiskers side, whiskers front, forelimbs, trunk, tail). Each row represents one recording session, and each column corresponds to a specific body part. Colors indicate the normalized change in SWR activity relative to baseline: blue denotes a decrease, and red denotes an increase. Statistical significance, assessed by Wilcoxon signed-rank test, is indicated by asterisks (*p < 0.05; **p < 0.01; Wilcoxon signed-rank test). (B) Same as (A) for movements for multiple body parts during delta-on epochs. Download Figure 4-2, TIF file. [file eneuro-12-ENEURO.0012-25.2025-s004.tif]
